# Leveraging basecaller’s move table to generate a lightweight k-mer model for nanopore sequencing analysis

**DOI:** 10.1093/bioinformatics/btaf111

**Published:** 2025-03-14

**Authors:** Hiruna Samarakoon, Yuk Kei Wan, Sri Parameswaran, Jonathan Göke, Hasindu Gamaarachchi, Ira W Deveson

**Affiliations:** School of Computer Science and Engineering, University of New South Wales, Sydney, NSW 2052, Australia; Genomics and Inherited Disease Program, Garvan Institute of Medical Research, Sydney, NSW 2010, Australia; Centre for Population Genomics, Garvan Institute of Medical Research and Murdoch Children’s Research Institute, Sydney, NSW 2010, Australia; Genome Institute of Singapore, A*STAR, Singapore 138672, Singapore; Yong Loo Lin School of Medicine, National University of Singapore, Singapore 117597, Singapore; School of Electrical and Information Engineering, University of Sydney, Sydney, NSW 2008, Australia; Genome Institute of Singapore, A*STAR, Singapore 138672, Singapore; Department of Statistics and Data Science, National University of Singapore, Singapore 117546, Singapore; National Cancer Center of Singapore, Singapore 168583, Singapore; School of Computer Science and Engineering, University of New South Wales, Sydney, NSW 2052, Australia; Genomics and Inherited Disease Program, Garvan Institute of Medical Research, Sydney, NSW 2010, Australia; Centre for Population Genomics, Garvan Institute of Medical Research and Murdoch Children’s Research Institute, Sydney, NSW 2010, Australia; Genomics and Inherited Disease Program, Garvan Institute of Medical Research, Sydney, NSW 2010, Australia; Centre for Population Genomics, Garvan Institute of Medical Research and Murdoch Children’s Research Institute, Sydney, NSW 2010, Australia; St Vincent’s Clinical School, Faculty of Medicine, University of New South Wales, Sydney, NSW 2052, Australia

## Abstract

**Motivation:**

Nanopore sequencing by Oxford Nanopore Technologies (ONT) enables direct analysis of DNA and RNA by capturing raw electrical signals. Different nanopore chemistries have varied k-mer lengths, current levels, and standard deviations, which are stored in “k-mer models.” In cases where official models are lacking or unsuitable for specific sequencing conditions, tailored k-mer models are crucial to ensure precise signal-to-sequence alignment, analysis and interpretation. The process of transforming raw signal data into nucleotide sequences, known as basecalling, is a fundamental step in nanopore sequencing.

**Results:**

In this study, we leverage the move table produced by ONT’s basecalling software to create a lightweight *de novo* k-mer model for RNA004 chemistry. We demonstrate the validity of our custom k-mer model by using it to guide signal-to-sequence alignment analysis, achieving high alignment rates (97.48%) compared to larger default models. Additionally, our 5-mer model exhibits similar performance as the default 9-mer models another analysis, such as detection of m6A RNA modifications. We provide our method, termed *Poregen*, as a generalizable approach for creation of custom, *de novo* k-mer models for nanopore signal data analysis.

**Availability and implementation:**

*Poregen* is an open source package under an MIT license: https://github.com/hiruna72/poregen.

## 1 Introduction

Nanopore sequencing allows for the direct examination of native DNA (Simpson *et al.* 2017, Zhang *et al.* 2023), RNA (Jain *et al.* 2022), and protein molecules (Hu *et al.* 2021), supporting many avenues of research across the life sciences. Instruments developed by Oxford Nanopore Technologies (ONT) detect changes in ionic current as these biomolecules traverse a nanoscale protein pore embedded in a charged membrane. The device captures time-series current signal data (Wang *et al.* 2021). To extract meaningful biological information from nanopore signal data, the data are typically first converted into DNA/RNA sequence reads through a process known as “basecalling.” Basecalling utilizes advanced algorithms, often involving neural networks, to identify and assign nucleotide bases based on the specific signal patterns (Wick *et al.* 2019). Signal data may also be analyzed directly to identify signatures from molecular features beyond the primary nucleotide sequence, such as DNA or RNA modifications (Wang *et al.* 2021).

Signal analysis and interpretation typically depends on the alignment of the raw electrical signal data with their corresponding nucleotide sequences. K-mers, short nucleotide sequences of a defined length (e.g. 5-mers), are used to guide the alignment process. Event alignment aims to match basecalled k-mers to their corresponding “events”—specific current levels observed in the raw signal that represent k-mers passing through the nanopore at different times. The necessary information is represented in a “k-mer model” (sometimes also referred to as the pore model or table), which is a simple table summarising the expected current level and variance associated with each possible k-mer, for a given nanopore type. Different nanopore chemistries exhibit distinct pore specifications, resulting in variations in the appropriate k-mer lengths, current levels, and standard deviations recorded in k-mer model. K-mer models assume a specific length for k-mers, which may or may not precisely match the actual k-mer length within the nanopore (Ding *et al.* 2021). Typically, a basic k-mer model includes $4^{k}$ different k-mers, where “*k*” represents the length of the k-mer, reflecting the four nucleotides (A, C, G, T/U) in DNA/RNA. Each k-mer, treated as an event, is associated with an expected current level and a standard deviation, capturing the variability in current levels observed for different k-mers passing through the nanopore. Various signal alignment methods, such as *Nanopolish/F5c event-align* (Simpson *et al.* 2017, Gamaarachchi *et al.* 2020), *Uncalled4*event-align (Kovaka *et al.* 2024), *Nanopolish* signal projection, *Sigmap* signal mapping (Zhang *et al.* 2021), and *Sigfish* dtw (Shih *et al.* 2023), rely on these k-mer models for accurate alignment of raw signal data to their corresponding nucleotide sequence. Signal alignment methods are vital in downstream analysis pipelines (Simpson *et al.* 2017). For example, the m6A modification detection tool—*m6Anet* (Hendra *et al.* 2022) uses the signal alignment produced by *Nanopolish/F5c*. The k-mer model may also be utilized during detection of modified nucleotides or other features, where observed signal data diverge from expected values for canonical bases in the model. Therefore, k-mer models are a critical element of nanopore signal data alignment and analysis.

It is important to note that while all bases within a k-mer influence the current or voltage level at a given moment, their contributions may vary. This variability in contribution is accounted for in the k-mer model, allowing for a more nuanced understanding of how different nucleotide combinations affect the observed electrical signals during nanopore sequencing.

Creating a *de novo* k-mer model is useful, especially in scenarios where an official model is not readily available or optimized for specific sequencing contexts. Usually, when ONT releases a new sequencing pore type, they also provide a corresponding k-mer model (https://github.com/nanoporetech/kmer\_models). However, the methods used to derive this k-mer model is proprietary (https://github.com/nanoporetech/remora) and recent instances, such as RNA004, saw delays in the release of their k-mer models. Without a suitable k-mer model, event alignment algorithms reliant on such models are ineffective or less reliable. This emphasizes the value of being able to create a tailored k-mer model from scratch, to facilitate accurate signal alignment and interpretation.

Moreover, the official ONT k-mer models often have exact k-mer lengths, leading to large models due to the exponential growth of possible k-mers (e.g. RNA004 with a 9-mer model has 4^9^ possible k-mers). Deducing lightweight k-mer models that maintain similar performance metrics is advantageous for computational efficiency and resource utilization.

Here, we describe a new method called *Poregen*, which we use to create a lightweight *de novo* k-mer model for ONT’s RNA004 chemistry. *Poregen* utilizes outputs from ONT basecalling software to empirically determine expected signal values and variances that make up a k-mer model. ONT basecalling software use Connectionist Temporal Classifiers (CTCs) to produce crude signal-to-base alignments (Graves *et al.* 2006). For example, in handwritten images, CTCs map sequential data such as strokes or characters to their image counterparts, ensuring accurate recognition (Zhan *et al.* 2017). Similarly, in basecalling, CTCs help align event data from raw signals to their basecalled sequences (Oxford Nanopore’s Basecaller—dorado 2024 https://github.com/nanoporetech/dorado). This alignment output is stored in a “move table,” which provides a crude mapping of signal events to their corresponding basecalled sequences. This move table provides the basis for our *Poregen* method. We gather a substantial number of samples for each k-mer using information from the move table and then calculate the mean and standard deviation. To ensure the quality of our model, we use various filtering techniques to capture only the most reliable samples. This manuscript outlines the *Poregen* method in detail, and provides performance metrics and comparisons with existing models to confirm the validity of the *de novo* k-mer model created.

## 2 Materials and methods

### 2.1 Data preparation

**Figure 1.**
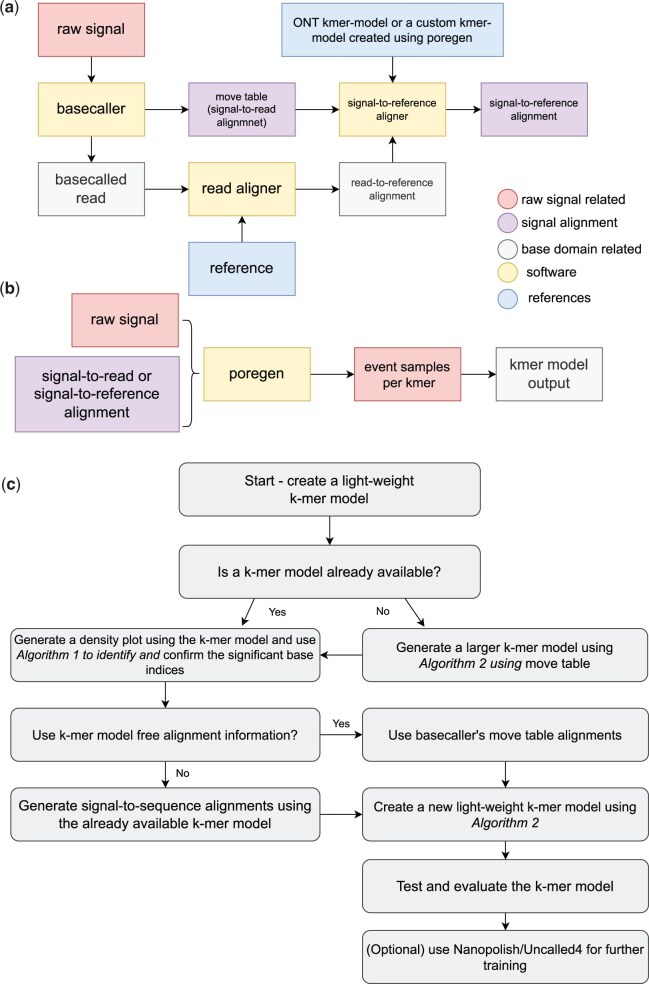
Schematic diagram summarizes data preprocessing steps and alternative workflow paths to generate custom k-mer models using *Poregen*. (a) The raw signal is basecalled and aligned to the reference. The move table is a signal-to-read alignment that does not depend on a k-mer model. Alternatively, the user may perform signal-to-reference alignment with a variety of external software, including *F5c, Nanopolish* or *Uncalled4* which in most cases require a k-mer model. (b) The signal-to-sequence encoded using “ss” format is filtered and fed to *Poregen* which does the sampling of k-mers. Then the mean and standard deviation are calculated for each k-mer to create a new k-mer model. (c) Flowchart outlining the process for creating a light-weight k-mer model. It involves either using an existing k-mer model or a move table to generate density plots for identifying significant base indices. *Poregen* accepts the basecaller’s move table or signal alignments generated using the existing k-mer model as input. The resulting model is evaluated and can optionally be refined further using *Nanopolish/Uncalled4*.

Our methodology for k-mer model creation uses a custom program called *Poregen*. This tool extracts current samples for each k-mer based on a provided alignment, which can either be a signal-to-read alignment (e.g. a move table generated by ONT basecalling software) or a signal-to-reference alignment (e.g. generated by *Nanopolish/F5c event-align*) (Fig. 1). In cases where a k-mer model for a specific nanopore chemistry is unavailable, *Poregen* can utilize the crude event alignment information in the ONT move table (Fig. 1). These alignments can be derived directly from signal-to-read mappings or reformatted signal-to-reference alignments generated using *Squigualiser Reform* or *Realign* subtools (Samarakoon *et al.* 2024).

*Poregen* requires three main inputs: raw signal data in SLOW5 format (Gamaarachchi *et al.* 2022, Samarakoon *et al.* 2023), sequences in FASTA format—either for basecalled reads or a reference genome/transcriptome—and signal-to-sequence alignments in SAM or PAF formats. The *Poregen* example command below is used to extract raw signal events for all 5-mers of a RNA dataset. By default, up to 5000 event samples are collected for each k-mer. Filtering is applied to retain only those events with lengths between 20 and 40 signal points. The signal is converted to pA values and normalized using Median-Median Absolute Deviation (Med-MAD) scaling. The scaled k-mer model is later transformed to real-world pA values (see Section 2.3):

poregen gmove reads.blow5 event-alignment.paf output_dir

--fastq reads.fastq -k 5 --sample_limit 5000 --rna

--min_dur 20 --max_dur 40 --scaling med-mad

The required signal-to-sequence alignment format is denoted as *ss* format (Samarakoon *et al.* 2024), representing the relationship between signal samples and the sequence. For example, the string “ss: Z:7,2D3,4I,5” translates to seven consecutive signal matches, two base deletions, three signal matches, four signal insertions, and five final matches along the sequence. Alignment tools such as *Nanopolish/F5c* and *Squigualiser* can output alignments in this format.

To ensure quality data, raw datasets should first be filtered based on metrics such as basecalling quality score (*qscore*), read-to-reference alignment score (*mapq*), and read length. These metrics establish a baseline for data reliability prior to processing with *Poregen*.

During event sampling, *Poregen* applies several additional filtering strategies to refine raw signal events:

- Dwell Time Thresholds: Events with durations outside a specified range are excluded to avoid noise. For example, the RNA004 chemistry has an average transition speed of 130 bases per second at a 4000 Hz sampling rate, resulting in ∼30 signal points per base. A dwell time range of 20–40 signal points provides a reasonable margin.
- Standard Deviation Filtering: Events with excessive standard deviation, indicating instability, are discarded.
- Indel Skipping (Optional): For signal-to-reference alignments, regions around insertions or deletions (indels) can be excluded, reducing the impact of noisy indel-associated signal variations. The number of skipped positions can be specified by the user, with a default value of 2bps.
- Sample Size: The number of event samples collected per k-mer is a critical parameter. While a sample size as low as 100 was sufficient in specific experiments (e.g. RNA004), *Poregen* defaults to collecting 5000 samples per k-mer to ensure statistical robustness.

These preprocessing and refinement steps allow *Poregen* to generate high-quality input data for constructing k-mer models tailored to various nanopore chemistries and experimental conditions.

Algorithm 1.
Finding the significant base indices of a k-mer model

1: **Input:** k-mer_model, k-mer_length, num_bins = 10, threshold = 0.96

2: **Result:** array with the significant base indices

3: 1-mer_models = []    ▹ array to store the 1-mer models

4: **for** base_index in k-mer_length **do**

5:  local_1-mer_model = {A:[], C:[], G:[], T:[]}  ▹ map to store the 1-mer model

6:  **for** k-mer, current_level in k-mer_model **do**

7:   base = k-mer[base_index]  ▹ pick base from {A, C, G, T}

8:   local_1-mer_model[base].append(current_level)  ▹ append current_level to base in the 1-mer model

9:  **end for**

10:  1-mer_models.append(local_1-mer_model) ▹ save 1-mer model

11: **end for**

12: significant_indices = [] ▹ indices of significant discrimination level

13: **for** base_index in k-mer_length **do**

14:  1-mer_model = 1-mer_models[base_index]

15:  similarities = []   ▹ array to store the per base similarities

16:  **for** base_i in {A, C, G, T} **do**

17:   x = 1-mer_model[base_i]

18:   **for** base_j in {A, C, G, T} - base_i **do**

19:    y = 1-mer_model[base_j]

20:    bins=num_bins evenly spaced numbers between (x, y)

21:    histograms=create histograms for x and y based on *bins*

22:    similarity=calculate Pearson value between histograms

23:    similarities.append(similarity)

24:   **end for**

25:  **end for**

26:  **if** mean(similarities) < threshold **then**

27:   significant_indices.append(base_index)  ▹ calculate the average similarity value for the 1-mer model and filter using the threshold

28:  **end if**

29: **end for**

30: **Return:** significant_indices

Algorithm 2.
Creating a new k-mer model

1: **Input:** move_tables, fasta_sequences, raw_signals, k-mer_length, num_samples, move_offset, min_dur, max_dur

2: **Result:** k-mer model

3: k-mer_model_mean = {}  ▹ store k-mer model as a maps

4: k-mer_model_stddev = {}

5: k-mer_counter = {}   ▹ count k-mer observations

6: **for** read in fasta_sequences **do**

7:  k-mer=fetch k-mers in sliding window from the read

8:  move_table = move_tables[read]

9:  raw_signal = raw_signals[read]

10:   move_table=move_table[move_offset:] ▹ apply move_offset

11:  **for** move in move_table **do**

12:   event=extract the corresponding region from the raw_signal

13:   **if** k-mer_counter[k-mer] < num_samples and min_dur < len(event) < max_dur **then**

14:    k-mer_model_mean[k-mer].append(mean(event))

15:    k-mer_model_stddev[k-mer].append(stddev(event))

16:   **end if**

17:   k-mer_counter[k-mer] += 1  ▹ Update k-mer_counter

18:  **end for**

19: **end for**

20: **for** k-mer in k-mer_model_mean **do**

21:  Calculate mean/median/stddev for final k-mer model

22:  k-mer_model_mean[k-mer]=mean(k-mer_model_mean[k-mer])

23:  k-mer_model_stdev[k-mer]=mean(k-mer_model_stddev[k-mer])

24: **end for**

25: significant_indices=Algorithm1(k-mer_model_mean, k-mer_length)    ▹ optional-check for smaller k-mer model

26: **Return:** k-mer_model_mean, k-mer_model_stddev

### 2.2 Identifying the most significant bases within a k-mer to create a lighter k-mer model

While all bases within a k-mer contribute to the observed current signal in nanopore sequencing, certain bases may have a stronger influence. Identifying these significant bases helps create light-weight k-mer models for improved efficiency. *Squigualiser’s calculate_offset* subtool, in combination with Algorithm 1, can be used to determine the most significant bases within a k-mer (Fig. 1c). Once these significant bases are identified, the k-mer length is recalculated to best capture the most significant base for every event. Typically, the k-mer length equals the count of significant bases. This approach allows *Poregen* to generate reduced k-mer models that focus only on these bases, significantly reducing the size of the k-mer model (see Section 2.3).

The Algorithm 1 is designed to identify significant base indices within a k-mer model. It takes a k-mer model as the input and starts creating 1-mer models for each base index in the k-mer (lines 3–11). Each 1-mer model is essentially a sub-model that captures the current levels corresponding to the bases A, C, G, and T for the given index. These 1-mer models store the current levels associated with each base (A, C, G, T) and each 1-mer has $4^{k}-\mathrm{mer}\_\mathrm{length}-1$ current levels. The goal of creating these 1-mer models is to isolate the contribution of individual bases at each position in the k-mer.

The next phase of the algorithm involves comparing these 1-mer models. It calculates the Pearson correlation between the histograms of current levels for different 1-mer pairs of a 1-mer model (lines 12–24). If the average similarity value for a 1-mer model falls below a specified threshold (0.96), the corresponding base index is considered significant (lines 26–27). This enables the algorithm to identify the positions in the k-mer where individual bases exert a stronger influence on the current signal.

Below are example commands for detecting significant base indices and visualizing them:

**#finding the significant base indices within a k-mer model**

  **as explained in**Algorithm 1

**squigualiser calculate_offsets --rna --use_model --model**

**RNA004_ONT_9mer.model--calculate_significant_indices**

**#visualizing the significant base indices**

**as a density plot using a k-mer model**

**squigualiser calculate_offsets --rna --use_model --model**

**RNA004_ONT_9mer.model --output model_density_plot.pdf**

### 2.3 Generating a new k-mer model

The generation of a new k-mer model is outlined in Algorithm 2. The algorithm processes each read and iterates through the bases in the alignment (line 6). For each k-mer, it extracts a user-specified number of current samples (lines 11–15), where each sample represents a single event consisting of a series of current values. The length of the event is further filtered using minimum and maximum duration thresholds (*min_dur* and *max_dur*) to ensure the inclusion of only well-defined signal events (line 13).

For each k-mer, the algorithm accumulates the current samples and then calculates either the mean (or median) and standard deviation [or median absolute deviation (MAD)] of the samples to generate the k-mer model (lines 20–23). This statistical representation ensures robustness against outliers and variability in the raw signals. Additionally, the user can specify a sufficiently large k-mer length (e.g. *k-mer_length *=* *9) to create an initial comprehensive k-mer model in cases where no pre-existing model is available (Fig. 1c). Once this initial model is built, significant bases can be identified using Algorithm 1 to refine the k-mer model further (line 25).

The Med-Mad normalized k-mer model is transformed into real-world pA values using the dataset’s global mean and standard deviation, following the formula: pA value = (normalized_current_mean × global_stddev) + global_mean. Since the normalized standard deviation values span a broader range, they are scaled to fit within the heuristic interval [2.5–4]. ONT and *Uncalled4* RNA004 k-mer models only have the current level mean values.

### 2.4 Evaluating a new k-mer model

There is no single definitive approach to evaluate the validity and/or performance of a k-mer model. In this study, we assess k-mer models using five complementary approaches, listed in order of their rigor in directly evaluating either the k-mer model itself or the signal alignment it produces:

1. Correlation analysis: We calculate the Pearson correlation coefficient between the current mean values of the new k-mer model and those of an existing k-mer model. A higher correlation suggests greater accuracy of the k-mer model. This can only be performed in cases where an existing model is available.
2. *Nanopolish/F5c* read alignment rate: The alignment rate is derived from summary statistics provided by the *Nanopolish/F5c event-align* program. This is calculated as the ratio of aligned reads to the total number of reads, expressed as a percentage. A higher alignment rate indicates superior model performance.
3. Visual Inspection via *Squigualiser* plots: We visualize signal alignments using *Squigualiser*, comparing alignments produced by the new k-mer model against those generated by the existing model.
4. Pipeline Accuracy Evaluation: We assess the accuracy of the final output from a downstream pipeline (e.g. an m6A detection pipeline) that incorporates event alignment data derived from the k-mer model.
5. F1 score metric: A new metric developed here, the F1 score evaluates one-to-one alignment mappings against a ground truth (in this case, alignments from an existing k-mer model) and the query (in this case, alignments from the new *de novo* k-mer model). We define the components as follows:

  • True Positives (TP): Signal points correctly mapped to the reference base. We allow a threshold of 1 base when determining true positives, meaning a signal point may be mapped to a base up to one position away (left or right) from the correct base and still be considered a TP.
  • False Positives (FP): Signal points incorrectly mapped to a base.
  • False Negatives (FN): Missed mappings that should have aligned to the reference base.
  • True Negatives (TN): Signal points that should not map to any base.
  • Precision, recall, and F1 score are calculated as: Precision = TP/(TP + FP), Recall = TP/(TP + FN), F1 Score = 2 × (Precision × Recall)/(Precision + Recall)

## 3 Results

We first analyzed the density plots generated using *Squigualiser’s calculate_offsets* subtool in conjunction with the Algorithm 1 to determine significant base indices of each official k-mer model available from ONT (see Section 2). Each subplot in Supplementary Figs S1–S5 illustrates the ability of each base position within the k-mer to discriminate between the four nucleotides (A, C, G, and T/U). Table 1 presents the most significant base indices obtained using the Algorithm 1. The density plots and indices corroborate each other’s findings. For instance, in the ONT 5-mer (r9.4.1 DNA) model, the last four bases emerge as the most significant indices, a finding echoed in the ONT 6-mer (r9.4.1 DNA) model. The ONT 5-mer (r9.4.1 RNA) model, on the other hand, exhibits significance across all its bases, with its density plot showing strongest significance around the central base and diminishing toward the ends (Supplementary Fig. S3).

**Table 1.** Significant base indices (0-based) for different k-mer models.

k-mer model	Significant indices	Density plot
ONT 5-mer (r9.4.1 DNA)	[1, 2, 3, 4]	Supplementary Fig. S1
ONT 6-mer (r9.4.1 DNA)	[1, 2, 3, 4]	Supplementary Fig. S2
ONT 5-mer (r9.4.1 RNA)	[0, 1, 2, 3, 4]	Supplementary Fig. S3
ONT 9-mer(r10.4.1 DNA)	[1, 2, 4, 5, 6, 7]	Supplementary Fig. S4
ONT 9-mer (RNA004)	[2, 3, 4, 5, 6]	Supplementary Fig. S5

The ONT 9-mer (r10.4.1 DNA) model displays two sets of most significant base indices (1,2 and 4,5,6,7), affirming the presence of the two reader heads within the pore. The recently published model for RNA004, ONT’s latest chemistry, is a 9-mer model, with most significant base indices at 2,3,4,5,6. We therefore reasoned that this 9-mer model may be reducible to a more lightweight 5-mer model capturing only the most significant bases, without compromising its analytical performance. An important rationale for this is that the 9-mer model contains 256 times more k-mers and occupies a memory footprint ∼300 times larger than the 5-mer model. Unnecessary inflation of the k-mer size may negatively impact analysis performance, as well as consuming excessive compute resources.

### 3.1 Evaluating a lightweight *de novo* model for RNA004 data

We used *Poregen* to generate a new *de novo* 5-mer (RNA004), based on a dataset from the Universal Human Reference RNA reference sample, and assessed the validity of the model via the approaches described above (see Section 2). Table 2 presents the Pearson correlation scores between each RNA model. In order to calculate the correlation between the 5-mer and 9-mer models, 9-mers were collapsed to their central 5-mers by taking their mean current value. All models were well correlated, with our *de novo* 5-mer model exhibiting similar current signal values to both ONT’s published 9-mer (RNA004) and 5-mer (r9.4.1 RNA) models, thereby validating our decision to opt for a 5-mer model.

**Table 2.** Mean current level correlation between k-mer models.

	ONT 9-mer (RNA004)	*F5c* 9-mer (RNA004)	ONT 5-mer (r9.4.1 RNA)	*Poregen* 5-mer (RNA004)
	A	B	C	D
A	1			
B	0.99898	1		
C	0.98417	0.98314	1	
D	0.99359	0.99462	0.97949	1

**Figure 2.**
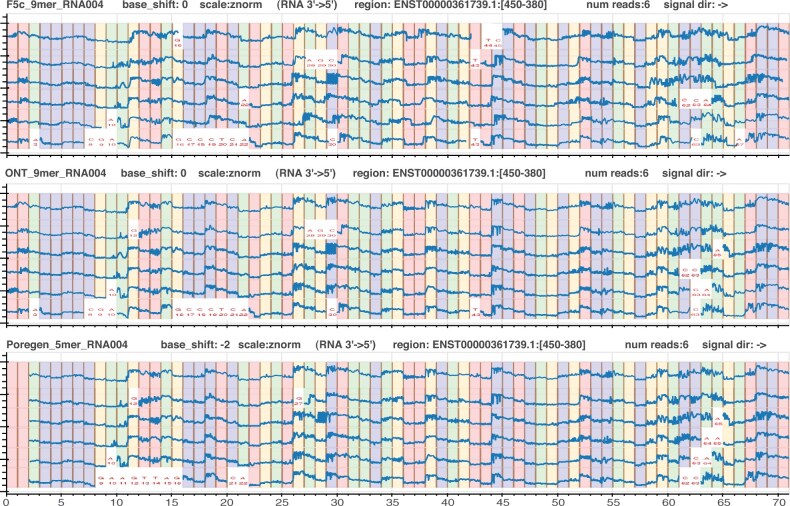
Visualization of *F5c event-align* output using three *Squigualiser* pileups for the same six RNA004 reads. The k-mer models used for each *event-align* step are, in order: (i) *F5c’*s built-in 9-mer (RNA004), (ii) ONT’s default 9-mer (RNA004), and (iii) a custom 5-mer (RNA004) model generated by *Poregen*.

We next evaluated the performance of each model during signal-to-reference alignment with *Nanopolish/F5c*, utilizes a k-mer model to guide alignment. The success of alignment is directly influenced by the accuracy of the k-mer model used (Table 3). The *F5c* event-alignment statistics reveal that all RNA004 models, including the *de novo* 5-mer model generated by *Poregen*, achieve alignment rates well exceeding 97% and F1 scores above 90%. In contrast, the r9.4.1 DNA 5-mer model, with a 0% alignment rate, confirms the alignment process is negatively impacted by use of an ineffective model (Table 3). Qualitative assessment of signal-to-reference alignments was also performed by visual inspection of aligned signal data at single-base resolution using *Squigualiser* (see Fig. 2). Signal pileups showed highly consistent event alignments across all three RNA004 models, further confirming the suitability of the *de novo* 5-mer model for signal alignment.

**Table 3.** *F5c* event-alignment statistics.

Model	Total entries	QC fail	Not calibrate	No alignment	No. of aligned reads	Alignment rate (%)	F1 score
*F5c* 9-mer (RNA004)	324 081	1665	5313	669	316 434	97.64	
ONT 5-mer (r9.4.1 RNA)	324 081	1813	4925	2671	314 672	97.10	0.92
ONT 9-mer (RNA004)	324 081	1534	5302	924	316 321	97.61	0.95
*Poregen* 5-mer (RNA004)	324 081	917	5319	1920	315 925	97.48	0.93
ONT 5-mer (r9.4.1 DNA)	324 081	73	461	323 512	35	0.00	0

**Figure 3.**
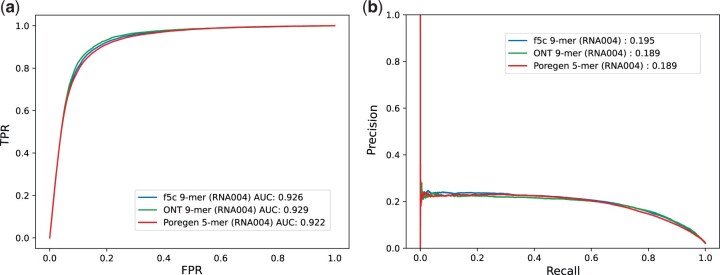
(a) ROC curves. (b) Precision-Recall curves of the m6A prediction performance of the k-mer models

Signal-to-sequence alignment typically precedes further signal-level analysis, such as detection of modified nucleotides. We therefore compared the performance of the available k-mer models for RNA004, including our custom model, during N6-methyladenosine (m6A) profiling analysis with *m6Anet* (Hendra *et al.* 2022). We used *F5c event-align* to align the current signal of a HEK293T RNA004 sample from the Singapore Nanopore Expression project (Chen *et al.* 2021). Each event-alignment output was used to predict the presence of m6A in the sample with *m6Anet’s* inbuilt RNA004 neural network model. We determined the m6A prediction performance using the m6ACE-seq-detected m6A sites as ground truth (see Table 4 and Fig. 3). We obtained superior performance with the *de novo* 5-mer model from *Poregen* compared to ONT’s 9-mer model, with more than twice the number of sites detected. The *Poregen* 5-mer also performed similarly to the inbuilt *F5c* 9-mer model. In summary, the *Poregen* method was able to produce a *de novo* 5-mer model for ONT RNA004 chemistry, that exhibited performance similar or superior to ONT’s published 9-mer model during signal alignment and RNA modification profiling analysis.

**Table 4.** Comparison of m6A sites detected.

Model	m6ACE sites	non-m6ACE sites
*F5c* 9-mer (RNA004)	16 899	748 524
ONT 9-mer (RNA004)	8102	380 903
*Poregen* 5-mer (RNA004)	16 996	762 264

### 3.2 Comparison with *Uncalled4* k-mer model generation method

*Uncalled4* (Kovaka *et al.* 2024) is an independent method developed in parallel to *Poregen*, which can also be used to generate a *de novo* k-mer model from information in the ONT basecaller move table. Instead of directly accessing the move table, *Uncalled4* uses the signal-to-reference alignment obtained using its own bcDTW algorithm. In addition to the current mean and current standard deviation the mean dwell time of each k-mer is also calculated in this method. *Uncalled4* has also generated and released a 9-mer model for RNA004. We next assessed the performance of this model against the *de novo* 5-mer model created by *Poregen*, by using each model to guide signal-to-reference alignment of matched data. We used the *Uncalled4 align* method to execute the alignments, and compared to alignments generated by *F5c event-align* as the ground truth. To confirm the compatibility of both methods with other external tools, we also used *Sigfish—*another tool that performs signal alignment using Dynamic Time Warping—guided by *Poregen* versus *Uncalled4* k-mer models. As summarized in Table 5, the *Uncalled4* 9-mer model and *Poregen* 5-mer model exhibited highly similar performance, with both showing F1 scores >90% regardless of which alignment method was used. This indicates both methods are valid and broadly compatible, and further confirms the feasibility of deriving a *de novo* k-mer model from data in the ONT move table.

**Table 5.** *Uncalled4* 9-mer and *Poregen* 5-mer models performance.

Query alignment (tool + model)	Ground truth alignment (tool + model)	F1 score
*F5c* + *Uncalled4* 9-mer	*F5c* + *F5c* inbuilt 9-mer	0.977
*F5c* + *Poregen* 5-mer	*F5c* + *F5c* inbuilt 9-mer	0.965
*Uncalled4* + *Poregen* 5-mer	*Uncalled4* + *Uncalled4*9-mer	0.979
*Uncalled4* + *Uncalled4*9-mer	*F5c* + *F5c* inbuilt 9-mer	0.925
*Uncalled4* + *Poregen* 5-mer	*F5c* + *F5c* inbuilt 9-mer	0.920
*Sigfish* + *Uncalled4* 9-mer	*Sigfish* + *Sigfish* inbuilt9-mer	0.975
*Sigfish* + *Poregen* 5-mer	*Sigfish* + *Sigfish* inbuilt9-mer	0.959

### 3.3 Generalizability and durability

To show that the *Poregen* method is generalizable to other nanopore chemistries, we next generated a *de novo* k-mer model for the alternative (now deprecated) direct RNA sequencing chemistry from ONT, RNA r9.4.1, using an available dataset and no guidance from the previously published k-mer model for RNA r9.4.1. The *Poregen* method was applied exactly as per the above approach for RNA004 data, with the only difference being that different mean and standard deviation values, calculated based on the available data, are used to transform and normalize the k-mer model to real-world pA values (see Section 2). To confirm the validity of the *de novo* RNA r9.4.1 5-mer models, we evaluated using the *F5c event-align* method, as above. It performed on par with the *F5c* inbuilt k-mer models (Table 6), confirming their validity.

**Table 6.** Performance of the Porgen model generated for r9.4.1 RNA.

Model	Total entries	QC fail	Not calibrate	No alignment	Alignment (%)	F1 score
*F5c* inbuilt 5-mer	17 142	767	110	90	94.359	–
*Poregen* 5-mer	17 142	490	180	565	92.795	0.914

This analysis illustrates how the same method can be used to create new *de novo* k-mer models for different nanopore types. While the k-mer model created is specific to a given pore type, it is largely unaffected by other experimental variables. This means a single k-mer model is suitable for analysis and interpretation of datasets from different experiments, protocols, and devices where the same pore-type was used. For example, the RNA004 *Poregen* 5-mer model was trained using a PromethION dataset. We used the same model to align a MinION dataset with *F5c event-align*, with signal data from the two devices having a range of different experimental variables and parameters, which are summarized in Table 7. Despite these differences, we observed good signal alignment performance with the MinION dataset, including an alignment rate of 97% and an F1 score of 94% (Table 8). These metrics confirm that the *Poregen* 5-mer model performed well in a different sequencing context, being largely comparable between MinION and PromethION data. This demonstrates that, while a new k-mer model should be generated when working with a different pore type (e.g. r9.4.1 versus RNA004), a single model is suitable for analysis of diverse datasets generated using the same pore type.

**Table 7.** Sequencing context-related parameters.

Parameter	PromethION dataset	MinION dataset
@asic_temp	47.121487	50.09399
@device_type	promethion	minion
@experiment_type	rna	rna
@flow_cell_product_code	FLO-PRO004RA	FLO-MIN004RA
@heatsink_temp	33.12566	34.320312
@protocols_version	7.7.6	7.8.2
@base speed per sec	130	130
@sequencing_kit	sqk-rna004	sqk-rna004
#digitisation	2048	8192
#range	299.432068	1437.976685

**Table 8.** Performance of the RNA004 PromethION dataset based Porgen 5-mer model on MinION dataset.

Model + dataset	Total entries	QC fail, not calibrate, no alignment	Rate (%)	F1score
*F5c inbuilt 9-mer* + PromethION	324 081	1665 + 5313 + 669	97.64	–
*Poregen* 5-mer + PromethION	324 081	917 + 5319 + 1920	97.48	0.934
*F5c inbuilt 9-mer* + MinION	228 196	53 + 1668 + 820	98.89	–
*Poregen* 5-mer + MinION	228 196	18 + 1712 + 2984	97.93	**0.940**

**Figure 4.**
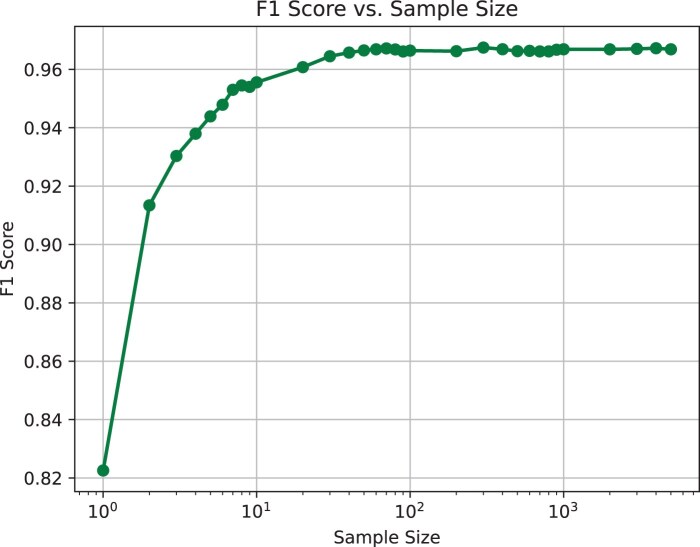
The F1 score measured for the RNA004 5-mer model increases with sampling depth up to ∼100. The performance reaches a maximum of ∼0.967 at this depth, with no significant improvement beyond it.

The depth of input data used during training is another variable that may affect the quality and robustness of a *de novo* k-mer model generated with *Poregen*. Since *Poregen* is a statistical method the number of events collected for a specific k-mer is important. To measure the ideal sampling depth, we reiterated the k-mer model creation process at different input data sizes and evaluated the resulting models based on alignment performance with *F5c event-align* (as above). Figure 4 demonstrates that the model performance increases with the sampling depth up to a maximum F1 score of ∼0.967 at around ∼100 depth—that is, when each k-mer has been seen ∼100 times. There is no improvement beyond this mark. This suggests a sample depth of 100 is sufficient for generating a good quality *de novo* k-mer model. However, a very conservative sample size of 5000 is set as the default in the *Poregen* program. Importantly, the size of the input data required to meet the minimum threshold is quite small; only ∼20 thousand RNA004 reads in the case of our experiment.

## 4 Discussion

K-mer models describing the expected relationship between DNA/RNA subsequences and current signal levels in nanopore sequencing are crucial for accurate signal alignment and interpretation. Our study focused on developing a *de novo*, light-weight k-mer model for the RNA004 chemistry, using the basecaller’s move table with data cleaning, sampling techniques, and significant base identification to ensure model quality and effectiveness. A key finding was the determination of the optimal 5-mer length for RNA004, balancing computational efficiency and discriminatory power. This is a useful insight, especially in resource-constrained settings, facilitating efficient signal interpretation and alignment.

Our *Poregen* method provides a generalizable method to build a *de novo* k-mer model for a new pore type, where an existing k-mer model is not available. The same method can be used for different pore types—as we’ve shown above for r9.4.1 versus RNA004 chemistries—and no prior knowledge is required, with *Poregen* using information in the basecaller move table in a relevant dataset to empirically determine an appropriate signal value and standard deviation for a given k-mer. The k-mer model generated is specific to a given pore type but is robust to other experimental variables, meaning a single model can be used for analysis of data from different experiments, protocols and devices, where the same pore type was used.

One limitation of this approach is that the quality of the *de novo* k-mer model is impacted by the quantity and quality of the input data in the move table. We show above that a minimum sampling depth of ∼100 observations for every possible k-mer is required to achieve optimal performance, with no improvement beyond this threshold. Only a relatively small quantity of input data is required to meet this requirement; for the RNA004 dataset considered here, just ∼20 thousand reads (∼20 Mbases) was sufficient (Supplementary Table S1). Data quality is a more relevant concern, as errors or artifacts in the move table may negatively impact the resulting k-mer model. While a degree of noise is inevitable, these effects can be mitigated by filtering the input dataset based on read quality scores, alignment scores and read lengths, and further filtering is applied by *Poregen* at the signal-event level (see Section 2). These steps are designed to remove low quality reads, messy alignments at indel regions, and/or experimental artifacts affecting the move table, providing only the cleanest data for use in building the *de novo* pore model.

To further improve the accuracy and robustness of the *de novo* k-mer model from *Poregen*, a user may choose to further refine the model through an iterative process, by using it as a custom model in the signal-to-reference alignment process (e.g. *Nanopolish/F5c event-align*). The newly generated signal-to-reference alignment serves as the input for *Poregen* in a subsequent run. This allows *Poregen* to extract samples based on a potentially more accurate alignment, leading to a refined k-mer model. Optionally, the user can consider using the *Nanopolish* training subtool. This tool iteratively fits a Gaussian mixture model (GMM) to the events detected for each k-mer, potentially leading to a more robust k-mer model after each iteration.

Overall, our work contributes methodological insights that advance nanopore sequencing by enabling lightweight and effective k-mer models tailored to specific chemistries, even in the absence of official models. Our comparison to an independent method with *Uncalled4* (Kovaka *et al.* 2024) confirms both are valid, together demonstrating the feasibility of creating useful *de novo* k-mer models from unseen dataset and pore-types.

## Supplementary Material

btaf111_Supplementary_Data

## Data Availability

*Poregen* (https://github.com/hiruna72/poregen) is free and open source with an MIT license. The RNA004 dataset along with the bash scripts to reproduce the 5-mer model generation is available at zenodo.10966311. The PromethION dataset used for *F5c event-align* is available at ENA: ERR12997170. The MinION RNA004 dataset is available at ENA: ERR12997172. The r9.4.1 RNA dataset is available at SRR22888949. The F1 score metric calculation and the its usage are available under src/f1_score in the GitHub repository.

## Supplementary data

Conflict of interest: I.W.D. manages a fee-for-service sequencing facility at the Garvan Institute of Medical Research and is a customer of Oxford Nanopore Technologies but has no further financial relationship. H.G. and I.W.D. have previously received travel and accommodation expenses from Oxford Nanopore Technologies. J.G. received reimbursement for travel and accommodation from Oxford Nanopore Technologies to present at the Nanopore Community Meeting in San Francisco in 2018. The authors declare no other competing financial or nonfinancial interests.
