# Supplementary material for: Leveraging basecaller’s move table to generate a lightweight k-mer model for nanopore sequencing analysis
Source: Bioinformatics. 2025 Mar 14;41(4):btaf111. doi: 10.1093/bioinformatics/btaf111 (PMC11964489; doi:10.1093/bioinformatics/btaf111)
Supplement: btaf111_Supplementary_Data [file btaf111_supplementary_data.zip › Supplementary Note 1.pdf]

# Supplementary Note 1: Leveraging basecaller's move table to generate a lightweight k-mer model for nanopore sequencing analysis

Hiruna Samarakoon, Yuk Kei Wan, Sri Parameswaran, Jonathan Göke, Hasindu Gamaarachchi, Ira W. Deveson

March 6, 2025

Table S1: Computer specifications and resource usage of *Poregen*

| Description | Value |
|---|---|
| System name | Workstation with SSD |
| CPU (No. of cores) | 2 x Intel(R) Xeon(R) Silver 4114 (40) |
| RAM | 384 GB |
| File system (Type) | SSD (ext4) |
| OS | Ubuntu 18.04 LTS |
| Reads per second (kbps) | ∼109 (127.35) |
| Peak RAM usage | 0.3 GB |
| 5-mer model generation with 20k reads | 3.5 minutes |

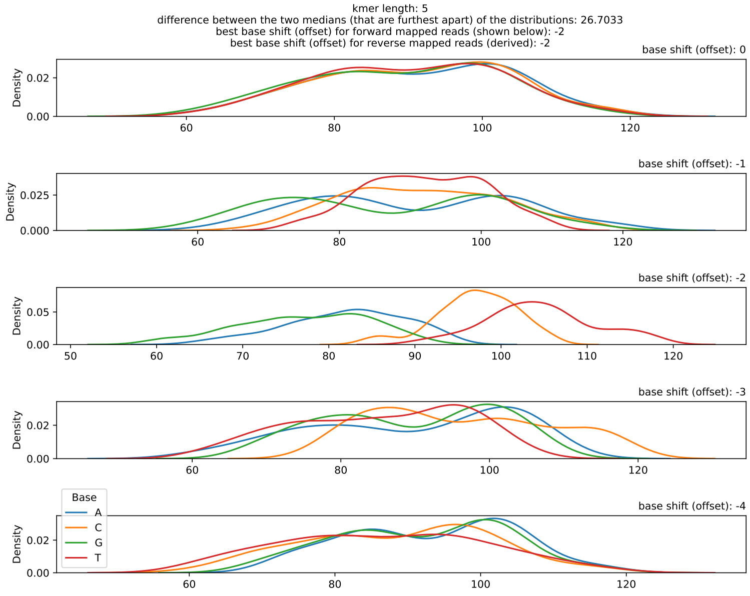


FigS1: ONT 5-mer (r9.4.1 DNA) model

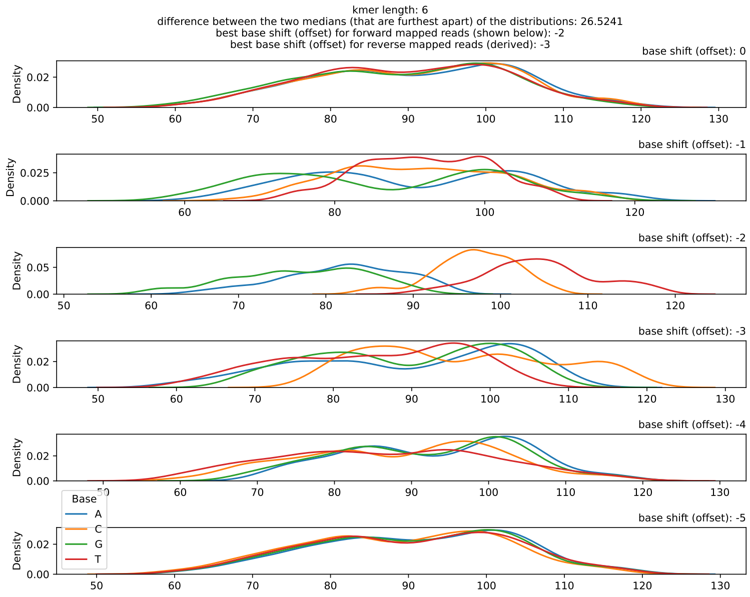


FigS2: ONT 6-mer (r9.4.1 DNA) model

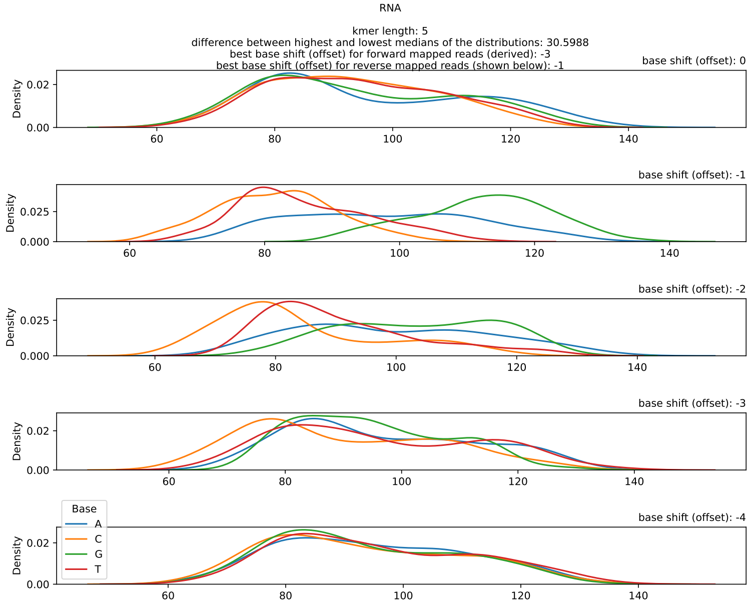


FigS3: ONT 5-mer (r9.4.1 RNA) model

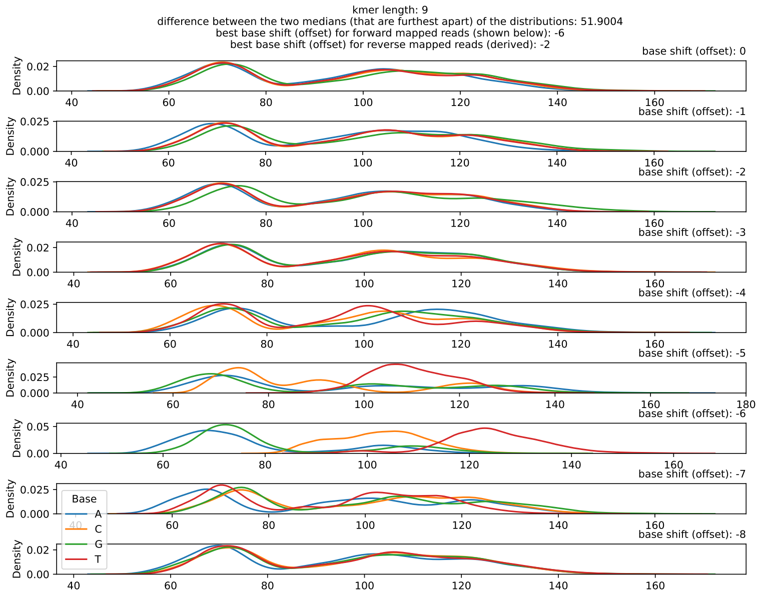


FigS4: ONT 9-mer (r10.4.1 DNA) model

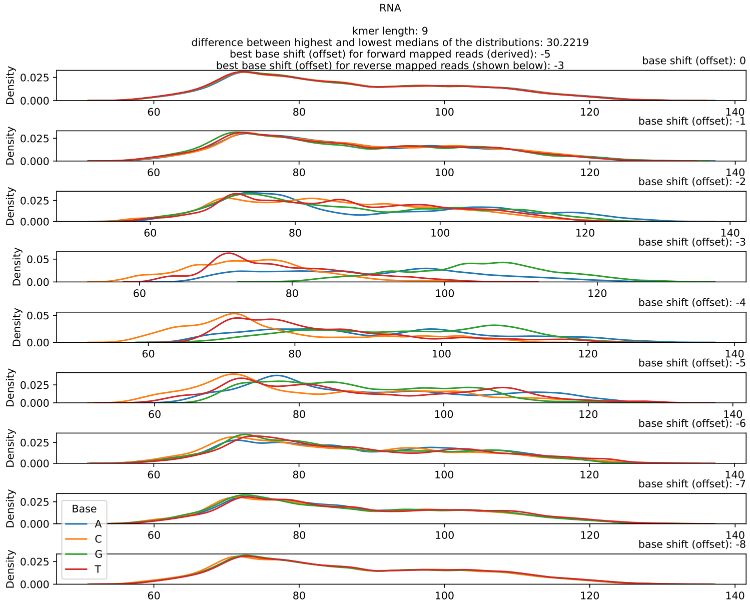


FigS5: ONT 9-mer (RNA004) model
